# The Association Between Essential Tremor and Parkinson’s Disease: A Systematic Review of Clinical and Epidemiological Studies

**DOI:** 10.3390/jcm14082637

**Published:** 2025-04-11

**Authors:** Elan D. Louis

**Affiliations:** 1 Department of Neurology, University of Texas Southwestern Medical Center, Dallas, TX 75390-8813, USA; elan.louis@utsouthwestern.edu; Tel.: +1-214-648-3571; 2 Peter O’Donnell Jr. Brain Institute, University of Texas Southwestern Medical Center, Dallas, TX 75390-8813, USA

**Keywords:** essential tremor, Parkinson’s disease, association, epidemiology, clinical, systematic review

## Abstract

**Background/Objectives:** The objective is to systematically review evidence from clinical and epidemiological studies for or against an association between essential tremor (ET) and Parkinson’s disease (PD). **Methods:** A literature search in PubMed (February 2025) used several combinations of keywords. Thirty-three studies (1960–2023) were identified. **Results:** The best available data are derived from a population-based study in Spain, followed by a cohort study in the US. Each of these prospective studies provided evidence that ET is a risk factor for PD, with elevated risks of ~4–5. In cross-sectional studies, in which the proportion of PD cases with ET has been reported, the weight of evidence demonstrates an association between ET and PD. In 16 (88.9%) of 18 family studies, the odds ratios or hazards ratios are elevated—i.e., there is considerable evidence that ET is over-represented in PD families and, conversely, PD is over-represented in ET families. **Conclusions:** A comprehensive review of published data strongly supports an association between ET and PD and, more specifically, provides evidence that ET is a risk factor for PD. Seven of nine review articles (and six of seven non-commissioned review articles) have concluded that there is an association between these two degenerative diseases. The “controversy” that surrounds the ET–PD association is more of a repeated myth than a well-informed reality. As a field, it would be more productive to finally move beyond uniformed debate and focus our efforts on attempts to elucidate the basis for the association to which the data are repeatedly pointing.

## 1. Introduction

### 1.1. Essential Tremor

Essential tremor (ET) is a chronic, progressive neurological disease [1], the primary clinical feature of which is kinetic tremor of the upper limbs [2], i.e., a tremor occurring during voluntary movements, but which may be accompanied by a range of other forms of tremor, other motor features and non-motor features [3,4,5,6]. The prevalence across all ages = 1.33%; the prevalence increases markedly with age, to approximately 6% in individuals age ≥ 65 years, and greater than 20% in some studies enrolling individuals in their 90s [7]. The incidence rate is similarly high—619 per 100,000 person-years in individuals age ≥ 65 years [8]. A recent study calculated an annual cost for direct medical care of ET patients age ≥ 40 years to be approximately USD 9.4 billion [9].

### 1.2. Parkinson’s Disease

Parkinson’s disease (PD) is a neurological disease that is chronic and progressive. The primary clinical features are tremor, bradykinesia, rigidity, and impaired postural reflexes [10]. The prevalence among persons aged 45 and older in north America has been estimated to be 0.572% [11], and the incidence is between 5 and 346 cases per 100,000 person-years [10]. The aggregated annual estimated direct medical expenditures of PD are approximately between USD 7.4 and USD 8.6 billion (2020) [9,12,13].

### 1.3. Sixth Sense of an Association Between ET and PD

For many years, movement disorders neurologists have noted that a certain proportion of ET patients seem to convert each year to ETPD (i.e., having both diseases) and that this proportion seems to be higher than expected due to chance alone [14,15,16]. In other words, ET seems to be a risk factor for incident PD. Furthermore, the association is specific to ET (i.e., there is no observed relationship between tics, chorea, or dystonia and subsequent development of PD).

### 1.4. Directionality of the Putative Association

The relationship between ET and PD seems to be a unidirectional one. That is, anecdotal observation and published data support the notion that ET leads to ETPD, whereas there are no data to support the notion that PD leads to PDET. A more detailed discussion of this topic may be found in Louis and Ottman [17]. One legitimate question, however, is whether it is possible to diagnose ET in a patient with PD, particularly when that patient has a tremor-predominant PD phenotype. There are numerous phenotypic differences between the action tremor of ET and that of PD [3,5,18,19,20], and head and voice tremors are common in ET but not in PD [21], indicating that there are many means by which to differentiate the action tremor of ET from that of PD. Nonetheless, it is possible that future research that explores the incidence of ET among PD patients (there are none at present) could underestimate any putative association.

### 1.5. Framing the Question—Is There an Association?

The aim of this paper is to systematically review evidence to support or refute an association between ET and PD. The most robust evidence is derived from longitudinal epidemiological studies of the incidence of PD in ET cases (i.e., that studies addressing whether or not ET is a risk factor for PD), but I also reviewed cross-sectional studies of the prevalence of ET (and in some cases action tremor) in PD cases and, conversely, of PD in ET cases, as well as studies of the familial aggregation of the two disorders.

## 2. Materials and Methods

A literature search in PubMed was conducted in February 2025, using several combinations of keywords. The combinations of these keywords yielded the following results: “Essential tremor” and “Parkinson’s disease” and “epidemiology” (215 publications), “Essential tremor” and “Parkinson’s disease” and “association” (176 publications), “Essential tremor” and “Parkinson’s disease” and “relationship” (109 publications), “Essential tremor” and “Parkinson’s disease” and “link (37 publications), ““Essential tremor” and “Parkinson’s disease” and “risk” (230 publications), and “Essential tremor” and “Parkinson’s disease” and “odds ratio” (30 publications). I excluded studies if they were not on humans or if they had an abstract that was not published in the English language. I reviewed in detail the studies that provided data on the incidence of PD in ET, the prevalence of PD in ET, the prevalence of ET (or action tremor) in PD, or the familial aggregation of the two disorders. I also reviewed the reference sections of these articles for additional studies pertinent to this review topic and obtained articles from my personal collection.

## 3. Results

### 3.1. Reviews

Apart from the current review, eight review articles have focused on the evidence for or against an association between ET and PD. To a variable degree, these studies have reviewed data on the incidence of PD in ET, the prevalence of PD in ET, the prevalence of ET (or action tremor) in PD, and the familial aggregation of the two disorders (Table 1) [15,21,22,23,24,25,26,27].

The first of these review articles was published in 1993 [15]. Four of the articles were published by the same author (initial article in 2007) [22] with periodic updates as new data emerged (2011 [21], 2015 [28] and 2019 [26]). Of these eight studies, six were derived from three author groups, who concluded that there was evidence for an association. One of the studies that concluded that there was no association was the earliest of these studies, reviewing data from six papers that were then available, and not including any prospectively collected data [15]. The other study that opined that there was no association only reviewed data from two studies [24].

In summary, seven of nine studies (including the current) have concluded that there is an association between ET and PD, including each of the four studies that reviewed 10 or more primary papers, each of the five studies that documented their search strategy, and all studies published since 2012. The only two studies that did not conclude that there was an association were as follows: an early study (now more than 20 years old) that reviewed six primary papers (and none of the prospective studies) and a point–counterpoint piece that was designed to conclude that there was no association and which reviewed only two primary papers.

### 3.2. Primary Literature

Review papers have limitations, one of which is that their year of publication reflects the state of the then-current literature. Degree of completeness and rigor also influence the quality of a review. Therefore, it is preferable to evaluate the primary literature itself. The ultimate aim of this review is to determine whether an ET diagnosis increases the risk of developing ETPD. Hence, prospective, longitudinal studies are of the greatest value. Retrospective, longitudinal studies are also of value, although are subject to all of the limitations that characterize studies with a retrospective design. Cross-sectional studies are of less direct value in addressing the primary question, although they can provide collateral evidence to support a causal association between the two disorders.

#### 3.2.1. Primary Literature—Prospective Longitudinal Studies

There are two prospective, longitudinal studies (Table 2) [29,30]. The value of these studies is that they did not simply assess the concurrent prevalence of PD in a group of ET patients but rather the incidence of PD (i.e., the subsequent conversion to ETPD) in that group of ET patients. This is to say that these studies focused on the evolution of a diagnosis/diagnoses rather than simple co-occurrence of two diagnostic entities [30].

The first, a prospective, population-based cohort study conducted in central Spain, estimated the incidence of PD in individuals whose baseline diagnosis was either ET or normal control (Table 2) [29]. The median follow-up interval was 3.3 years, but it was as long as 6.6 years in some of the cases [29]. During this time period, 6 (3.0%) of 201 ET cases versus 24 (0.7%) of 3574 controls developed incident PD (unadjusted relative risk [RR] = 4.34, 95% confidence interval [CI] = 1.77–10.62, p = 0.001; adjusted RR = 4.27, 95% CI = 1.72–10.61, *p* = 0.002) [29]. The mean latency between the onset of ET and the onset of incident PD in these six ET cases was 8.7 (median 8.0, range 5.6–12.7) years [29]. A variety of sensitivity analyses were performed by the investigators to evaluate the robustness of their findings, and the results of these analyses yielded similar risk estimates [29]. The sensitivity analyses related to the following issues: method of ET diagnosis, duration of ET diagnosis, and method of PD diagnosis [29]. The investigators estimated that the lifetime risk of developing PD was 8.5% in men with ET and 5.6% in women with ET, compared with 2.0% in men and 1.3% in woman without ET [29]. A limitation of the study is that the procedure to diagnose PD involved a two-stage process, and it is possible that some PD cases may have been misclassified as controls; however, as noted by the authors, this potential source of misclassification would likely have biased their results towards the null hypothesis [29].

A second prospective cohort study was conducted in the United States (Table 2) [30]. Enrollment was nationwide, with patients being geographically widespread rather than being ascertained from a treatment setting [30]. This reduced a bias associated with treatment settings, that is, the tendency to enroll complex cases and/or cases with multiple concurrent neurological diagnoses [30]. An additional strength is that all ET and PD diagnoses were assigned by a senior movement disorders neurologist. A design limitation was that there was no control group; hence, the authors used data from historical controls [30]. The median follow-up interval was 4.6 years [30]. During this time period, seven (3.6%) of 193 ET cases converted from ET to ETPD [30]. Hence, the incidence of PD among patients with ET was 882.8/100,000 person years (py) [30]. The duration of ET at the time of conversion to PD was 8–79 years (median = 65.0). In a meta-analysis of non-ET cases (i.e., controls) in 14 studies from 13 countries across 4 continents, the authors noted that the incidence of PD in men peaked in the 80- to 89-year-old age group (258.47/100,000 py) and in women in the 80- to 89-year-old age group (103.48/100,000 py) [30]. The authors concluded that the risk of PD in ET is 2–6.5 times higher than expected in the general population [30].

Several caveats should be discussed. First, in prospective studies, it is important to ensure that the initial ET diagnosis is valid because early PD may manifest as an isolated action tremor. Steps to mitigate that risk include careful phenotyping (e.g., the isolated action tremor of PD is more often a unilateral postural tremor than a bilateral kinetic tremor) as well as a stipulation that the ET diagnosis precede the PD diagnosis by a reasonable time period, which is often set at 5 years [30]. Second, it is important that the diagnosis of PD in ETPD is based not only on the presence of rest tremor [30]. This is because patients with longstanding and severe ET may develop rest tremor [32].

In sum, two prospective cohort studies provided data on the high incidence of PD in ET cases, and both have reported a similar magnitude of elevated risk. While additional studies would provide data from other cohorts, the two available studies already provide construct validity for one another, as the direction and the magnitude of the enhanced risk are consistent across studies.

#### 3.2.2. Primary Literature—Retrospective Longitudinal Studies

There is one additional cohort study, although this used a retrospective rather than prospective design (Table 2) [31]. The primary purpose of that study was to estimate the incidence of ET in Rochester, Minnesota, and furthermore provide retrospective, longitudinal data on mortality in these cases compared to historical controls [31]. Data were ascertained from a broad time period, from 1935 to 1979, and were abstracted from medical record information during that time period. There are limited data on the presence or development of PD. Diagnostic criteria for ET and PD were not specified but were applied by an unstipulated number of physicians during this long time domain. The authors noted that a diagnosis of incident “Parkinsonism” was made in six (2%) ET cases after their index date [31]. Data on the completeness of ascertainment of PD is not specified, but serial Unified Parkinson’s Disease Rating Scale examinations were not part of the study design. Furthermore, data on the duration of the follow-up interval in converters and nonconverters were not provided; hence, incidence rates were not reported [31]. It is difficult to make inferences from these data [31].

#### 3.2.3. Primary Literature—Cross-Sectional Studies of the Prevalence of PD in ET Cases

A number of cross-sectional studies have reported the proportion of ET cases who also have PD (Table 3). It is important to note that these studies did not have a longitudinal design, as did those reviewed above. Second, as noted above, the proportion of complex ET cases (e.g., ET cases with other concurrent diagnoses such as PD) is heavily dependent on the ascertainment scheme, with clinics (and especially referral clinics) being more likely to ascertain a higher proportion of such cases. Despite the use of these studies to support or refute an association between ET and PD, the methodological limitations of many of these studies make the results uninterpretable with regard to the question at hand. These studies will be reviewed.

A paper in 1960 in rural Sweden provided data on 81 ET cases (Table 3) [33]. While one of the review papers cited above [15] reported that only one of these cases had parkinsonism and none had PD, this does not seem to be correct. Two cases had pill-rolling tremor and 15 had rigidity in one or more limb. Regardless, the ET diagnoses themselves are questionable. The study assessments were performed in the field in the mid-1950s, and not by movement disorders neurologists. Definitions of ET and PD are not clearly articulated. ET was characterized by “rest tremor”, although they likely meant postural tremor, and furthermore, the ET definition does not incorporate kinetic tremor, which is the primary feature of ET [2,38]. Oddly, 23 (28.4%) of these ET cases had tremor in the legs, which is a high proportion, further raising doubts about the underlying ET diagnoses. Finally, many of the ET cases were related to one another—these were tremor families in many instances. Although this study has been portrayed as one that does not support an association between ET and PD [15], if anything, the data point to a high prevalence of rigidity in ET cases. However, the study design features make the results difficult to interpret.

A study conducted in the United States in 1985 utilized data from 130 ET cases enrolled in a movement disorders clinic (Table 3) [34]. There was no control group. In total, 25 (19.2%) of 130 enrollees had both ET and ETPD. Although this proportion is high, these cases were enrolled from a tertiary referral clinic, and the high proportion of ETPD cases could have reflected an ascertainment bias. Indeed, an unspecified number of these ET cases had secondary diagnoses of ET, indicating that another diagnosis (e.g., PD) was the primary reason for ascertainment. The authors concluded that the prevalence of PD in ET was 24 times greater than expected, although they did not provide the calculation upon which this was based.

Another study in 1991 from the same center was a retrospective analysis of 350 ET patients seen at a movement disorders clinic (Table 3) [35]. The investigators noted that 71 (20.3%) of 350 ET cases had ETPD [35]. Although this proportion is high, the same issues of ascertainment bias and absence of a control group are applicable here as well, making the interpretation of findings difficult.

In a similar study in the United States published in 1994, 678 ET patients were ascertained from clinical settings (university and private practices), and 6.1% of these were found to have ETPD (Table 3) [36]. Many (371 or 54.7%) were ascertained from a national survey of neurologists who filled out a brief form about ET and PD diagnoses in their patients; hence, the methods for evaluating and diagnosing patients were highly heterogenous and likely often inexpert [36]. The same issues of ascertainment bias and absence of a control group are applicable here as well, making the interpretation of findings difficult [36].

A 1997 study of 357 ET patients ascertained from three hospitals in Spain reported that 31 (8.7%) also had PD (Table 3) [37]. The same issues of ascertainment bias and absence of a control group are applicable here as well, making the interpretation of findings difficult.

In sum, the cross-sectional studies discussed above are for the most part characterized by methodological limitations that make the interpretation of results challenging. In these studies, the proportion of ET cases with PD is quite high, and in some studies is close to or exceeding 20%. On face value, this appears to be higher than expected for age- and gender-matched controls. However, this apparent excess of PD cases could also be simply the result of ascertainment bias.

#### 3.2.4. Primary Literature—Cross-Sectional Studies of the Prevalence of ET in PD Cases

The cross-sectional literature on the prevalence of ET in PD is presented in Table 4. In some instances, the authors reported the prevalence of action tremor rather than ET per se, making the information less valuable.

A French-Canadian study published in 1982 [16] proposed a classification system for PD. “Type IIIb”, termed “Familial ‘Essential-Tremor-Related’ Parkinsonism”, comprised PD families with dominant inheritance of ET as well. They wrote: “cases of PD appear more frequently in “essential tremor” families because these patients are genetically more susceptible to whatever triggers the appearance of PD”. In sum, the paper reports the existence of a high proportion of PD families in their region with both ET and PD (Table 4). While sometimes cited as a study that supports an association between ET and PD, the high proportion could be an artifact of the genetic milieu in which the study was conducted.

A study published in 1988 enrolled 100 PD patients from a clinic in London. Of these, 3 (3.0%) “had a postural tremor distinct from classical rest tremor and reported tremor as a first symptom” for 5, 14 and 22 years, respectively, before the onset of other PD symptoms (Table 4) [39]. Without a control group, it is difficult to interpret whether this proportion is higher than expected.

Importantly, one of the studies, from 2008, had a case-control design (Table 4) [41]. Patients were recruited from a tertiary referral center in Singapore, including 204 with PD, 206 diseased controls (i.e., neurological outpatients with hemifacial spasm) and 190 healthy controls (medical and technical staff from the hospital as well as volunteers) [41]. The authors reported that 12 (5.9%) PD patients had an additional diagnosis of ET, compared to 2 (1.7%) diseased controls (odds ratio [OR] = 6.4, 95% CI = 1.5–27.7, *p* = 0.006) and 1 (0.5%) healthy control (OR = 11.8, 95% CI = 1.9–71.3, *p* = 0.003) [41]. The data provided are of greater value than those in the other studies because they resulted in an OR. While the ascertainment from a clinic raises concerns about selection bias, the fact that the co-occurrence of ET in PD patients was greater than that of ET in another disease group makes that a more remote possibility.

There are an additional two studies that similarly demonstrate that the association between ET and PD is specific to PD and not a feature of other degenerative forms of parkinsonism. The first of these was a case-control study that was conducted in the United States (Table 4) [40]. The investigators reviewed electronic records of 210 PD patients and 210 patients with a Parkinson-plus syndrome, with the hypothesis that if there were a link between ET and Lewy body disease, one would observe that ET patients who go on to develop parkinsonism would be more likely to develop PD, a Lewy body disease, than a Parkinson-plus syndrome, a non-Lewy body form of parkinsonism [40]. They found that PD patients had a higher probability of a prior diagnosis of ET than patients with Parkinson-plus syndrome (7.1% vs. 2.4%; OR = 3.16, 95% CI = 1.13–8.85, *p* = 0.02) and had a higher likelihood of having had a diagnosis of ET that was assigned by a neurologist at their visit (5.3% vs. 0.0%; OR = 12.85, 95% CI = 1.66–99.80, *p* = 0.001) [40]. Second, in a paper published in abstract form in 2010 [42], medical records of consecutive patients with PD, progressive supranuclear palsy (PSP) and corticobasal degeneration (CBD) over a two-year period were analyzed (Table 4). Presence of ET-type tremor at least 5 years prior to the onset of the parkinsonian disorder was found in 13% of PD, 4% of PSP, and none of CBD cases.

In sum, there are five studies of the prevalence of ET in PD. One study, which had a case-control design, allowed for a direct comparison between PD cases and control groups, and that study reported that a higher-than-expected proportion of PD cases had carried diagnoses of ET. Two studies reported that the association was specific to ET and PD [40,42], rather than ET and parkinsonian disorders that are not associated with Lewy pathology. There are no studies that provide ORs that refute an association between ET and PD. Hence, the weight of these data supports an association between ET and PD.

#### 3.2.5. Primary Literature—Studies of the Familial Aggregation of the Two Diseases

Data from family studies offer a valuable perspective (Table 5).

In a population-based family study from Finland published in 1976 (Table 5) [43], 25 (5.8%) relatives of PD cases vs. 36 (8.1%) relatives of controls reported a family history of ET (*p* = 0.09). The study had a number of strengths, including the large sample size and use of a control group, but there were substantial weaknesses—relatives were not systematically examined in order to verify proband reports, diagnostic criteria for ET were not specified by the authors, and most importantly, no adjustment was made for differences in size and age distributions of PD and control families.

In the French-Canadian study published in 1982 [16], none of their controls had first-degree relatives with ET vs. 20% PD cases with tremor at onset and 8% of PD cases with rigidity/akinesia at onset (Table 5). Despite the apparent association between ET and PD, there are numerous methodological problems with this study: the method of eliciting a report of a diagnosis of ET is not entirely clear, and the diagnostic criteria for ET were not specified. Also, it is entirely possible that a proband with familial PD would be more likely to be aware of and report tremor in a family member than would a control proband. One interesting and unique feature of this study is the attempt to separate PD into different phenotypic subtypes.

In a study published by the same group in 1983 (Table 5) [44], the investigators studied 50 PD kindreds in whom the PD proband’s disease had started by age 40 years. In 28 (56.0%) of 40 kindreds, one or more relatives reportedly had ET; in 19 (38.0%) kindreds, two or more relatives reportedly had ET; in 8 (16.0%) kindreds, 4 or more relatives reportedly had ET; and in 4 (8.0%) kindreds, 8 or more relatives reportedly had ET. In the 50 kindreds with PD, there were 100 cases of ET (3.9% of the estimated 2550 relatives) vs. in the 50 control kindreds, in which 6 or 1803 (0.3%) estimated relatives reportedly had ET. While on the surface the results suggest an association between ET and PD (OR = 12.22, 95% CI = 5.35–27.92, *p* < 0.0001), there are numerous methodological problems with this study. Specifically, the number of relatives was estimated based on average family size in an unspecified sample of families, the method of eliciting a report of a diagnosis of ET is not entirely clear, and the diagnostic criteria for ET were not specified. Also, it is entirely possible that a proband with familial PD would be more likely to be aware of and report tremor in a family member than would a control proband.

In a 1984 study in Finland (Table 5) [45], the investigators queried probands (194 ET from an epidemiological study, 115 ET from clinic, and 125 controls) whether they had any family members with PD. They did not interview family members directly or examine the relatives, which are both limitations. Also, with each proband, they did not review the disease status of relatives one-by-one but rather simply asked the proband whether there was a family history of PD. They noted that the ET and control families were of similar size but provide no data on family size, number of potentially affected relatives, ages of the relatives, or any differences in the ages of the relatives between ET groups and controls. They also did not specify the proportions of first-degree relatives vs. second-degree relatives. In ET families, 2/173 (1.2%) (epidemiological study) and 0/108 (0%) (clinic) reported a first-degree relative with PD vs. 1/105 (0.9%) controls. In ET families, 4/173 (2.3%) and 3/108 (2.8%) vs. controls 4/105 (3.8%) reported any relative (first- and beyond) with PD. Although this study has been cited as one that disputes the association between ET and PD [22], one can see that the methodological rigor was low.

A 1987 study in Canada compared 159 PD probands with 104 control probands; 27 (17%) PD probands and only 6 (5.6%) control probands reported having a first-degree relative with isolated postural tremor (Table 5) [46]. Limitations of the study included the following: self-report data, data on “isolated postural tremor” rather than ET, and no adjustment for differences in family size or age in the two proband groups [46].

In a study published in the US in 1988 (Table 5) [39], the researchers used data from 237 ET patients attending clinics (137 in London and 100 in Chicago), 100 PD cases attending a clinic in London, and 100 spouses of patients (i.e., the control group). The criteria for ET are not rigorous (i.e., presence of postural or kinetic tremor of the hands or head without other neurological signs). The evaluation of relatives was primarily by self-report, unless there was a positive report, in which some of these were seen in person. The statistical design was unsophisticated; the investigators used chi-square tests to compare proportions rather than using Cox proportional hazards models to examine the relationship between the time-to-event outcome (i.e., development of ET in a relative) and an explanatory variable (neurological diagnosis of relative’s proband). The percentage of probands with a family history of PD was as follows: PD = 8.0%, ET = 3.8%, and controls = 6.0%. Furthermore, 16 (3.5%) of the 463 PD relatives (including parents and siblings) had “tremor” vs. 7 (1.5%) of 452 relatives of controls. After including children, 17 (2.6%) of the 650 PD relatives (parents, siblings, and children) had “tremor” vs. 7 (1.0%) of the 674 relatives of controls. The authors stated that the differences were not significant, but the respective ORs (calculated from their data) were 2.28, 95% CI = 0.93–5.59, *p* = 0.036 and 2.56, 95% CI = 1.05–6.21, *p* = 0.019. Additional problems with the study design include the fact that “tremor” is not ET and it is not well-defined, and there was no adjustment for family size or age of the relatives in PD vs. control families.

A family study in Finland in 1988 [47] enrolled 52 PD patients (Table 5). These patients reported the presence of ET in their parents and siblings. In the relatives, the incidence of ET per 1000 py was 2.03; this did not differ from the expected value for the incidence of ET (1.67), which was derived from a population-based study in Finland. The limitations of the study were substantial: the evaluation of relatives was almost exclusively by self-report and the diagnostic criteria for ET were not specified. Furthermore, there was no direct control group for comparison.

A study in Canada in 1993 selected PD cases from a population-based case register (Table 5) [48]. In total, 130 PD cases and 260 controls reported the family history of ET, and OR = 2.37, 95% CI = 1.20–4.69 (*p* < 0.01) [48]. A strength of the study was the enrollment of a control group, thereby allowing for the calculation of an OR. However, weaknesses included the following: the disease status in the relatives was by proband report only, diagnostic criteria for ET were not specified, and no adjustment was made for family size or age of the relatives in PD vs. control families [48]. The authors reported the same results in a paper published in 1995 [55].

A family study in Spain reported that 14.9% of 74 PD patients and 4.7% of 148 controls reported a family history of postural tremor (OR = 3.52, 95% CI = 1.19–10.62, *p* = 0.009) (Table 5) [49].

A study in Germany in 1994 reported that 13.6% of 66 PD cases vs. 4.2% of 72 controls reported a family history of ET (OR = 3.62, Table 5) [50]. Only the abstract is published in English.

A 1994 study in the UK of 20 familial ET cases reported that none of the 93 first-degree or 38 more distantly related relatives had PD (Table 5) [51]. A strength of the study was the examination of all relatives by a movement disorders neurologist. Although this has been cited as a paper that disputes the association between ET and PD [22], the small sample size and absence of a control group make these data difficult to interpret—there is no reported OR.

In a 1994 study at a university movement disorders clinic in the US (Table 5), 4.4% of parents and siblings of PD patients reported having isolated tremor vs. 1.0% of parents and siblings of controls (OR = 4.80, *p* = 0.0007) [52]. Furthermore, 18% of PD patients and 4% of controls had a family history of tremor in a first-degree relative (*p* = 0.002). Despite the large sample size, and the use of a control group, “isolated tremor” rather than ET was reported, and self-reports were not validated by in-person examinations of the relatives [52].

A family study in Italy in 1995 (Table 5) [53] reported that a positive family history of ET was noted in 17 (17%) of 100 PD patients, 5 (5%) of 100 spouses of PD patients (OR = 3.89 ^a^, 95% CI = 1.38–11.01, *p* = 0.005) and 6 (6%) of 100 controls (OR = 3.21, 95% CI = 1.21–8.52, *p* = 0.01). A strength of the study was the use of two controls groups. Limitations are similar to those of other studies discussed above (Table 5). In 1996, the authors reported similar results from an expanded sample—116 in each group (Table 5) [56].

A 1995 study in a movement disorders clinic in the US noted that tremor was reported in 96 (5.1%) of 1874 parents and siblings of PD patients, 12 of 462 (2.6%) such relatives of PSP patients, and 10 (2.3%) of 429 relatives of controls (OR for PD vs. controls = 2.26, 95% CI = 1.17–4.38, *p* = 0.008) [54]. The strengths and limitations of this study are noted in Table 5.

A study at an academic medical center in the US noted that a family history of tremor was reported in 12.1% of 140 PD cases and 3.4% of 147 controls (OR = 3.97, 95% CI = 1.17–13.50, *p* = 0.027). Study strengths included the large sample size and the enrollment of a control group. Limitations are similar to those of numerous other family studies (Table 5) [57].

In a study in a movement disorders center in Italy in 2002, a family history of ET in a first-degree relative was reported in 9 (6.6%) of 136 PD cases and 3 (1.1%) of 272 controls (OR = 10.8, 96% CI = 2.6–43.7, *p* = <0.0001, Table 5). Study strengths and limitations are similar to those of numerous other family studies (Table 5) [58].

A study of 162 PD patients in California in 2005 noted that 15.4% reported a positive family history of ET. There was no control group for comparison (Table 5) [59].

In a study in 2007, researchers in the Mayo Clinic Family Study of Parkinson’s Disease assessed the risk of ET in relatives of patients with PD (Table 5) [60]. They compared 981 first-degree relatives of 162 patients with incident PD to 838 first-degree relatives of 147 controls from the Olmsted county population [60]. The first-degree relatives for tremor were first screened using a telephone interview, and they then examined the relatives who screened positive [60]. For incapacitated or deceased family members, the study team used data from an informant. PD relatives had a marginally significantly increased risk of ET compared to control relatives (hazards ratio [HR] = 1.51, 95% CI = 0.95–2.41, *p* = 0.08), with the highest HR in relatives of PD patients whose onset was younger (HR = 2.24, 95% CI 1.26–3.98, *p* = 0.006) [60]. Study design limitations include the use of screening interviews and data from proxies [60]. However, an important element of this study is the use of a sophisticated statistical design—Cox proportional hazards models.

A familial aggregation study in 2009, conducted as part of the PD Epidemiology Program Project of Crete (Table 5) [61], assessed the presence of ET in first-degree relatives of 303 PD patients and 249 controls [61]. The investigators first obtained proband reports and then performed in-person evaluations in 78% of reportedly affected PD relatives and 70% of reportedly affected control relatives [61]. For those relatives who remained, medical records were used to confirm diagnoses, as were interviews with surrogates [61]. Twenty-six (8.6%) of 277 PD patients vs. 8 (3.2%) of 241 controls reported at least one first-degree relative with ET (OR = 2.83, 95% CI = 1.19–6.92, *p* = 0.015) [61]. Furthermore, 41 (2.8%) of 1483 first-degree relatives of PD cases had ET vs. 10 (0.8%) of 1315 first-degree relatives of controls (OR = 3.64, 95% CI = 1.75–7.77, *p* = 0.0001) [61]. 

In a population-based family study in California in 2010, investigators assessed the occurrence of ET in relatives of 372 PD patients and 404 controls (Table 5) [62]. They assessed 2980 first-degree relatives of PD cases and 2981 first-degree relatives of controls [61]. Proband interviews were used to assess family history [62]. The PD relatives had a modestly increased risk of having ET, although this increased risk was only marginally significant—45 of 2980 (1.5%) first-degree relatives of PD patients had ET vs. 31 of 2981 (1.0%) first-degree relatives of controls (HR = 1.44, 95% CI -= 0.90–2.29, *p* = 0.13) [62]. In a number of the subgroups of PD patients, the risk of ET was most elevated, including relatives of tremor-dominant PD cases (HR = 1.69, 95% CI 0.99–2.88, *p* = 0.05), relatives of PD cases with younger-onset (HR = 2.03, 95% CI = 0.93–4.44, *p* = 0.08) as well as relatives who were male (HR = 2.31, 95% CI = 1.13–4.73, *p* = 0.02) [62]. Limitations of the study design include the fact that diagnosis of ET in relatives was exclusively by proband report [62]. However, an important element of this study is the use of a sophisticated statistical design—Cox proportional hazards models.

In a study in the USA published in abstract form in 2010 (Table 5) [42], medical records of consecutive patients with PD, PSP and CBD were analyzed. Family history of ET-type tremor was identified in 21.5% of PD cases, 6.5% of PSP cases, and 5.0% of CBD cases [42]. The study has many of the same methodological limitations as those discussed above.

In a clinical-epidemiological study in the USA published in 2016, 110 probands with ET, 130 with PD, 27 with ETPD, and 177 control probands reported whether they had relatives with these diseases or with non-specific tremor (Table 5) [63]. A larger proportion of the probands with PD than the control probands reported relatives with PD (20.0% vs. 8.5%, *p* = 0.003) and ET (11.5% vs. 2.8%, *p* = 0.002) [63]. However, the proportion of ET probands and control probands who reported a relative with PD was similar (10.9% vs. 8.5%, *p* = 0.49) [63]. Limitations of the study design include the reliance on proband reports and the simple study design, which merely reported proportions [63].

In summary, numerous family studies have been conducted in order to gauge the presence of ET cases within PD families and vice versa. The studies have numerous limitations, although some study designs are more robust than others. Sixteen (88.9%) of 18 studies report elevated odds or risks, compared with 2 (11.1%) that do not report elevated odds or risks. The latter two derive from the same population-based study in Finland. Every study reporting ORs or HRs that was published after 1984 reports a positive association. Two studies use a more sophisticated study design, reporting HRs rather than ORs, thereby allowing them to examine the relationship between the time-to-event outcome (i.e., development of ET in a relative) and an explanatory variable (neurological diagnosis of relative’s proband)—these both report a significant association. When one assesses all the data, the over-arching result is that the ORs and HRs are elevated—that is, there is considerable evidence that ET is over-represented in PD families and that, conversely, PD is over-represented in ET families.

#### 3.2.6. Primary Literature—Synthesis

I have systematically assessed the evidence from a variety of primary studies. The best available data are derived from a population-based study in Spain [29], followed by a cohort study in the US [30]. Each of these prospective studies provide evidence that ET is a risk factor for PD, with an elevated risk on the order of 4–5. One retrospective, longitudinal study is difficult to interpret due to substantial limitations in study design [31]. In cross-sectional studies, the proportion of ET cases with PD is quite high, and in some studies is close to or exceeding 20%. On face value, this appears to be higher than expected for age- and gender-matched controls. However, this apparent excess of PD cases could also be simply the result of ascertainment bias. In cross-sectional studies, the proportion of PD cases with ET has been reported. One study had a case-control design, allowing for direct comparisons between PD cases and control groups, and that study reported that a higher-than-expected proportion of PD cases had carried diagnoses of ET. Two studies report that the association is specific to ET and PD [40,42], rather than ET and parkinsonian disorders that are not associated with Lewy pathology. The weight of these data supports an association between ET and PD. Numerous family studies have been conducted in order to gauge the presence of ET cases within PD families and vice versa, and these studies have generated numerous ORs and HRs. Sixteen (88.9%) of eighteen studies report elevated odds or risks, compared with two (11.1%) that do not report elevated odds or risks. The latter two derive from the same population-based study in Finland. Every study reporting ORs or HRs that was published after 1984 reports a positive association. In sum, the evidence in favor of an association between ET and PD is overwhelming and dwarfs any evidence that there is no association.

### 3.3. Supportive Biomarker Studies

As reviewed above, the weight of the clinical and epidemiological evidence supports an association between ET and PD. At this point, it is valuable to review related evidence. A number of studies have provided evidence of mild PD-like biological signals in ET cases, and even among ET cases without rest tremor or other clinical features of evolving ETPD. The studies suggest that PD is brewing within ET populations to a greater extent than within control populations. In a study that used transcranial sonography to assess substantia nigra hyperechogenicity, 74 PD cases differed from controls with respect to mean hyperechogenicity (14.53 ± 11.49 mm^2^ vs. 3.51 ± 3.47 mm^2^). Values in ET did not differ significantly from those of controls, although numerically were nearly twice as high (6.11 ± 8.26 mm^2^ vs. 3.51 ± 3.47 mm^2^) [64]. In a similar study of 44 ET patients and 100 controls, 16% of ET patients had substantia nigra hyperechogenicity (defined as an area of echogenic signal of 0.24 cm^2^ or greater on at least one side) vs. 3% of controls (*p* < 0.005) [65]. In a study involving single-photon emission computed tomography (SPECT) with 123I-ioflupane imaging of 28 ET patients and 28 controls, qualitative interpretation of the SPECT data did not find any difference in the uptake of the radiopharmaceutical at the level of the striatum, caudate nucleus, or putamen between ET patients and controls but semi-quantitative analysis demonstrated a reduction in striatal dopamine in the ET group [66]. In another DaTscan study enrolling 22 ET cases and 13 controls, striatal binding ratio was numerically slightly lower in ET than controls in the caudate (1.81 ± 0.29 vs. 1.92 ± 0.27 in right caudate, and 1.82 ±0.31 vs. 1.95 ± 0.34 in the left caudate), although this difference was not statistically significant [67]. A study of rapid eye movement sleep behavior disorder (RBD) in 64 ET patients reported that 5 (8.3%) had RBD, a proportion that was considered higher than expected in the general population [68]. Erythrocytic total α-synuclein levels and erythrocytic aggregated α-synuclein levels were found to be significantly elevated in a study of 15 ET cases compared to 49 controls (*p* <0.001 and *p* < 0.05, respectively) [69]. These types of biomarker studies provide some evidence, albeit limited, that ET appears on some level to be a cauldron for ETPD.

### 3.4. Mechanistic and Biological Underpinnings

Following on the epidemiological and biomarker data, the underlying mechanism whereby ET leads to an increased risk of ETPD should be considered. An obvious explanation is the increased burden of Lewy pathology in ET cases. A recent study, coming from an analysis of the largest repository of prospectively collected ET brains, reported that 58 (24.1%, i.e., nearly one-in-four) of 231 ET cases had Lewy pathology [70], a value that the authors calculated was 50–75% higher than that observed in studies of the prevalence of Lewy pathology in normal elderly individuals. It is of particular interest that two epidemiological studies report that the association of ET and parkinsonism is specific to ET and PD [40,42], rather than ET and other forms of parkinsonism that are not associated with Lewy pathology. Why ET cases develop such high levels of Lewy pathology, however, requires further investigation. On an etiological level, shared genetic risks or environmental risk factors could lead to both diseases. The current evidence for this is modest. In fact, most data do not support the notion that ET and PD share genetic risk factors [71,72,73]. A small number of studies support the notion that harmane, a dietary toxin, and lead are environmental risk factors both for ET and PD [74,75,76,77]; however, the data are modest at present. Moving downstream from etiological factors, one must consider the underlying brain substrate. The mechanisms that underlie PD are numerous, with genetic forms of PD linked to a variety of molecular mechanisms related to mitochondria, the autophagy-lysosomal system and vesicular transport, and alpha-synuclein aggregation [78,79]. Additional mechanisms implicated in PD include oxidative stress [80], neuroinflammation [81], as well as neuropeptides (e.g., orexin) [82], indicating a complex and heterogeneous disorder. To date, these mechanisms have not been linked to the pathophysiology of ET, although a number of these mechanisms have not been studied in ET as of yet, and would be worthwhile exploring in future studies. 

On a broad level, having one neurodegenerative disease increases the risk for others. Stated in another manner, propensity for neurodegeneration may manifest in different brain regions at different times and in different ways. There are numerous other examples of such [83,84,85,86]. Clearly the field is ripe for discovery. In the future, both the primary etiological factors in ET (i.e., genetic and/or environmental) and resultant molecular underpinnings will require further elaboration in clinical, epidemiological and basic science studies. Furthermore, additional postmortem studies of patients with both diseases, ET and PD, would be of value.

### 3.5. Conclusions

While it is often stated in the literature that the association between ET and PD is “controversial”, all but two of the review papers on this topic have arrived at the same conclusion—that there is an association. One of the two review articles that argued against the association was part of a point–counterpoint dyad and was commissioned to argue that there was no association; furthermore, that review included data from only two epidemiological studies. The second of these articles was an early article that reviewed data from six studies. More recent reviews have had the advantage of access to 30 or more studies, including two prospective, longitudinal studies. Hence, the weight of the review articles supports an association.

Reviewing the primary data is imperative. When reviewing the data, it is important to be cognizant of the strengths and weaknesses of study design, which in some cases limit the inferences that can be made. Not all data are of equal value.

In their sum, the primary data strongly support an association between ET and PD, and, more specifically, provide evidence that ET is a risk factor for PD. A biological basis, the increased Lewy pathology burden in ET, has been observed. The unidirectional nature of the relationship, as well as its specificity, is also intriguing and adds further weight to the argument that there is a bone fide association.

The “controversy” that surrounds the ET–PD association is more of a repeated myth than a well-informed reality. If a “controversy” means “people who are not fully cognizant of the data have opinions”, then there is a “controversy”. But that is not the same statement as “there is no credible evidence for an association” or “there is no association” or “the data are of such poor quality that we can make no conclusions” or “there are equally balanced data that demonstrate and refute a relationship”. In reality, when we put aside uniformed opinions, the data speak for themselves, and they show that the evidence for an association is robust.

As a field, it would be more productive to finally move beyond uniformed debate and to focus our efforts on attempts to elucidate the basis for the association to which the data are pointing.

## Figures and Tables

**Table 1:** Nine review articles on the association between ET and PD.

Author (Year)	PubMed Search Strategy Was Documented	Number of Primary Studies ^1^ Reviewed	Number of Prospective Studies ^2^ Reviewed	Final Conclusion (Association Vs. No Association)	
				Association	No Association
Pahwa and Koller (1993) [15]	No	6	0		×
Shahed and Jankovic (2007) [22]	No	11	0	√	
LaRoia and Louis (2011) [23]	Yes	6	1	√	
Fekete and Jankovic (2011) [21]	Yes	10	1	√ ^3^	
Adler et al. (2011) [24]	No	2	1		× ^3^
Jiménez-Jiménez et al. (2012) [27]	Yes	30	1	√	
Thenganatt and Jankovic (2015) [25]	No	7	1	√	
Tarakad and Jankovic (2019) [26]	Yes	8	1	√	
Current Study	Yes	33	2	√	

^1^ Studies that provided data on the incidence of ET in PD, the prevalence of PD in ET, the prevalence of ET (or action tremor) in PD, or the familial aggregation of the two disorders. ^2^ Studies that provided data on the incidence of PD in ET. ^3^ Part of a point–counterpoint dyad, designed to come to the conclusion it did. Jankovic was the author of four of these review articles.

**Table 2:** Primary literature—three longitudinal studies.

Author (Year)	Location	Design	Main Finding	Study Strengths	Study Limitations
Rajput et al. (1984) [31]	USA	Retrospective	A diagnosis of incident “Parkinsonism” was made in six (2%) ET cases after their index date, but there was no reported RR or HR.	Longitudinal design Large study sample	Data were ascertained over a broad time frame, from 1935 to 1979, and were abstracted from medical record information during that time period. Diagnostic criteria for ET and PD were not specified, but were applied by an unspecified number of physicians during this long time domain. Data on the completeness of ascertainment of PD are not specified, but serial Unified Parkinson’s Disease Rating Scale examinations were not part of the study design. Data on the duration of the follow-up interval in converters and nonconverters were not provided; hence, incidence rates were not reported.
Benito-Leon et al. (2009) [29]	Spain	Prospective	Adjusted RR = 4.27 (95% CI = 1.72–10.61, *p* = 0.002)	Population-based Prospective design Large sample size	Procedure to diagnose PD involved a two-stage process, and it is possible that some PD cases may have been misclassified as controls; however, as noted by the authors, this potential source of misclassification would likely have biased their results towards the null hypothesis.
Louis et al. (2023) [30]	USA	Prospective	The incidence of PD among patients with ET was 882.8/100,000 py, which was 2–6.5 times higher than expected in the general population.	Prospective design Large sample size Enrollment was nationwide, with patients being geographically widespread rather than being ascertained from treatment settings. This reduced a bias associated with treatment settings—enrollment of complex cases and/or cases with multiple concurrent neurological diagnoses. All ET and PD diagnoses were assigned by a senior movement disorders neurologist.	No control group; hence, the authors used data from historical controls.

CI = confidence interval, ET = essential tremor, HR = hazards ratio, PD = Parkinson’s disease, py = person-years, RR = relative risk.

**Table 3:** Primary literature—five cross-sectional studies of the prevalence of PD in ET cases.

Author (Year)	Location	Design	Main Finding	Study Strengths	Study Limitations
Larsson and Sjogren (1960) [33]	Sweden	Cross-sectional study of ET cases	In total, 2 (2.5%) of 81 ET cases had pill-rolling tremor and 15 (18.5%) had rigidity in one or more limb.	Population-based design	No control group The ET diagnoses themselves are questionable. The study assessments were performed in the field in the mid-1950s, and not by movement disorders neurologists. Definitions of ET and PD are not clearly articulated. ET was characterized by “rest tremor”, although they likely meant postural tremor, and the definition does not incorporate kinetic tremor. 23 (28.4%) of these ET cases had tremor in the legs, which is a high proportion, further raising doubts about the underlying ET diagnoses. Many of the ET cases were related to one another– these were tremor families in many instances.
Geraghty et al. (1985) [34]	USA	Cross-sectional study of ET cases	In total, 25 (19.2%) of 130 ET cases had both ET and ETPD. The authors concluded that the prevalence of PD in ET was 24 times greater than expected, although did not provide the calculation upon which this was based.	Large sample size	No control group As these cases were enrolled from a tertiary referral clinic, the high proportion of ETPD cases could have reflected an ascertainment bias.
Lou and Jankovic (1991) [35]	USA	Cross-sectional of ET cases	In total, 71 (20.3%) of 350 ET cases had ETPD.	Large sample size	No control group. Ascertainment from a clinic (i.e., ascertainment bias).
Koller et al. (1994) [36]	USA	Cross-sectional study of ET cases	In total, 6.1% of 678 ET cases had ETPD.	Large sample size	No control group. Ascertainment from a clinic (i.e., ascertainment bias). Many (371 or 54.7%) were ascertained from a national survey of neurologists who filled out a brief form about ET and PD diagnoses in their patients; hence, the methods for evaluating and diagnosing patients were highly heterogenous and likely often inexpert.
Tallon-Barranco et al. (1997) [37]	Spain	Cross-sectional study of ET cases	In total, 31 (8.7%) of 357 ET patients also had PD.	Large sample size	No control group. Ascertainment from a clinic (i.e., ascertainment bias).

ET = essential tremor, PD = Parkinson’s disease.

**Table 4:** Primary literature—five cross-sectional studies of the prevalence of ET in PD cases.

Author (Year)	Location	Design	Main Finding	Study Strengths	Study Limitations
Barbeau et al. (1982) [16]	Canada	Cross-sectional	PD cases appeared frequently in ET families.		No control group. The high proportion could be an artifact of the genetic milieu in which the study was conducted.
Cleeves et al. (1988) [39]	England	Cross-sectional	In total, 3 (3%) of 100 PD patients had postural tremor reported as a first symptom ≥ 5 years before the onset of other PD symptoms.		No control group. Ascertainment from a clinic (i.e., ascertainment bias).
Louis and Frucht (2007) [40]	USA	Cross-sectional enrolling several comparison groups (210 PD patients and 210 Parkinson-plus syndrome patients)	PD patients had a higher likelihood of a prior ET diagnosis than patients with Parkinson-plus syndrome (7.1 vs. 2.4%; OR = 3.16, 95% CI = 1.13–8.85, *p* = 0.02) and they more likely to have had a diagnosis of ET assigned by a neurologist at the time of their visit (5.3% vs. 0.0%; OR = 12.85, 95% CI = 1.66–99.80, *p* = 0.001).	Case-control design	ET was ascertained based on a retrospective medical records review.
Tan et al. (2008) [41]	Singapore	Cross-sectional enrolling several comparison groups (204 PD, 206 diseased controls, 190 healthy controls)	In total, 12 (5.9%) of 204 PD patients had an additional diagnosis of ET, compared to 2 (1.7%) of 206 diseased controls (OR = 6.4, 95% CI = 1.5–27.7, *p* = 0.006) and 1 (0.5%) of 190 healthy control (OR = 11.8, 95% CI = 1.9–71.3, *p* = 0.003).	Case-control design The study generated ORs.	
Fekete et al. (2010) [42]	USA	Cross-sectional enrolling several comparison groups (PD, progressive supranuclear palsy, corticobasal degeneration) but sample size not specified	Presence of ET-type tremor at least 5 years prior to the onset of the parkinsonian disorder was found in 13% of PD, 4% of progressive supranuclear palsy, and none of corticobasal degeneration.	Case-control design	Data only published in abstract form. “ET-type tremor” rather than ET was ascertained based on a retrospective medical records review. Sample size not specified.

CI = confidence interval, ET = essential tremor, OR = odds ratio, PD = Parkinson’s disease.

**Table 5:** Primary literature—twenty-four studies of the familial aggregation of the two diseases.

Author (Year)	Location	Design	Main Finding	Study Strengths	Study Limitations
Marttila and Rinne (1976) [43]	Finland	Population-based family study	Twenty-five (5.8%) of 429 relatives of PD cases vs. 36 (8.1%) of 443 relatives of controls reported a family history of ET (OR = 0.7 ^a^, 95% CI = 0.41–1.19, *p* = 0.09).	Population-based. Large sample size. Control group.	Relatives were not systematically examined to verify proband reports. Diagnostic criteria for ET were not specified. No adjustment for differences in size and age distributions of PD and control families.
Barbeau et al. (1982) [16]	Canada	Family study	In total, 20% of PD cases with tremor at onset, 8% of PD cases with rigidity/akinesia at onset, and 0% of controls had first-degree relatives with ET.	Stratification of PD probands into two groups. Control group.	The method of eliciting a report of a diagnosis of ET is not entirely clear. The diagnostic criteria for ET were not specified. A proband with familial PD might be more likely to be aware of and report tremor in a family member than would a control proband.
Roy et al. (1983) [44]	Canada	Family study	In 50 PD kindreds, there were 100 cases of ET (3.9% of the estimated 2550 relatives); in 50 control kindreds, 6 of 1803 (0.3%) estimated relatives reportedly had ET (OR = 12.22 ^a^, 95% CI = 5.35–27.92, *p* < 0.0001).	Data collected on a large number of relatives. Control group.	The number of relatives was estimated based on average family size in an unspecified sample of families. The method of eliciting a report of a diagnosis of ET is not entirely clear. The diagnostic criteria for ET were not specified. A proband with familial PD might be more likely to be aware of and report tremor in a family member than would a control proband.
Martilla et al. (1984) [45]	Finland	Family study	In ET families, 2/173 (1.2%) (from an epidemiological study) and 0/108 (0%) (from a clinic) reported a first-degree relative with PD vs. 1/105 (0.9%) in control families (OR for first group of ET cases vs. controls = 1.21 ^a^, 95% CI = 0.11–13.55, *p* = 0.87). In ET families 4/173 (2.3%) and 3/108 (2.8%) vs. controls 4/105 (3.8%) reported any relative (first- and beyond) with PD.	Large sample size. Control group.	They did not interview relatives directly or examine the relatives. With each proband, they did not review disease status of relatives one-by-one, but rather, simply asked the proband whether there was a family history of PD. They noted that the ET and control families were of similar size, but provide no data on family size, number of potentially affected relatives, ages of the relatives, or any differences in the ages of the relatives between ET groups and controls. They also did not specify the proportions of first-degree relatives vs. second-degree relatives.
Lang et al. (1987) [46]	Canada	Family study	In total, 27 (17%) of 159 PD probands vs. 6 (5.6%) of 104 control probands reported having a first-degree relative with isolated postural tremor (OR = 3.34 ^a^, 95% CI = 1.33–8.40, *p* = 0.005).	Large sample size. Control group.	Self-report data on disease status. Data were provided on “isolated postural tremor” rather than ET. No adjustment for differences in family size or age in the two proband groups.
Cleeves et al. (1988) [39]	USA	Family study	The percentage of probands with a family history of PD was as follows: PD = 8.0%, ET = 3.8%, and controls = 6.0%. Furthermore, 16 (3.5%) of the 463 PD relatives (including parents and siblings) had “tremor” vs. 7 (1.5%) of 452 relatives of controls. After including children, 17 (2.6%) of the 650 PD relatives had “tremor” vs. 7 (1.0%) of the 674 relatives of controls. The authors stated that the differences were not significant, but the respective ORs were 2.28 ^a^, 95% CI = 0.93–5.59, p = 0.036 and 2.56 ^a^, 95% CI = 1.05–6.21, *p* = 0.019.	Large sample size. Control group.	The criteria for ET are not rigorous (i.e., presence of postural or kinetic tremor of the hands or head without other neurological signs). The evaluation of relatives was primarily by self-report, unless there was a positive report, in which some of these were seen in person. The statistical design was unsophisticated; the investigators used chi-square tests to compare proportions rather than using Cox proportional hazards models to examine the relationship between the time-to-event outcome (i.e., development of ET in a relative) and an explanatory variable (neurological diagnosis of relative’s proband). “Tremor” was not well-defined and it is not the same as ET. There was no adjustment for family size or age of the relatives in PD vs. control families.
Martilla and Rinne (1988) [47]	Finland	Family study	In total, 52 PD patients reported the presence of ET in their parents and siblings. The incidence of ET per 1000 py was 2.03, but this did not differ from the expected value for the incidence of ET (1.67), which was derived from a population-based study in Finland.		The evaluation of relatives was primarily by report. The diagnostic criteria for ET were not specified. No control group.
Semchuk et al. (1993) [48]	Canada	Family study	In total, 130 PD cases and 260 controls reported the family history of ET (OR = 2.37, 95% CI = 1.20–4.69, *p* < 0.01).	Control group.	Disease status in the relatives was by proband report only. The diagnostic criteria for ET were not specified. There was no adjustment for family size or age of the relatives in PD vs. control families.
Morano et al. (1994) [49]	Spain	Family study	In total, 14.9% of 74 PD patients and 4.7% of 148 controls reported having a family history of postural tremor. The OR = 3.52, 95% CI = 1.19–10.62, *p* = 0.009.	Control group.	“Postural tremor” rather than ET was assessed.
Vieregge et al. (1994) [50]	Germany	Family study	In total, 13.6% of 66 PD cases vs. 4.2% of 72 controls reported a family history of ET–OR = 3.62.	Control group.	Only the abstract is published in English
Bain et al. (1994) [51]	United Kingdom	Family study	In 20 familial ET cases, none of the 93 first-degree or 38 more distantly related relatives had PD.	The examination of all relatives was performed by a movement disorders neurologist.	The small sample size and absence of a control group make these data difficult to interpret.
Payami et al. (1994) [52]	USA	Family study	4.4% of 586 parents and siblings of PD patients reported having isolated tremor vs. 1.0% of 522 parents and siblings of controls (OR = 4.80 ^a^, 95 CI = 1.83–12.60, *p* = 0.0007). Furthermore, 18% of PD patients and 4% of controls had a family history of tremor in a first-degree relative (*p* = 0.002).	Large sample size. Control group.	“Isolated tremor” rather than ET was reported in the relatives, and self-reports were not validated by in-person examinations of the relatives.
De Michele et al. (1995) [53]	Italy	Family study	A positive family history of ET was noted in 17 (17%) of 100 PD patients, 5 (5%) of 100 spouses of PD patients (OR = 3.89,^a^ 95% CI = 1.38–11.01, *p* = 0.005) and 6 (6%) of 100 controls (OR = 3.21 ^a^, 95% CI = 1.21–8.52, *p* = 0.01.	Large sample size. Two control groups.	Disease status in the relatives was by proband report only. The diagnostic criteria for ET were not specified. There was no adjustment for family size or age of the relatives in PD vs. control families.
Jankovic et al. (1995) [54]	USA	Family study	Tremor was reported in 96 (5.1%) of 1874 parents and siblings of PD patients, 12 of 462 (2.6%) such relatives of PSP patients, and 10 (2.3%) of 429 relatives of controls (OR for PD vs. controls = 2.26 ^a^, 95% CI = 1.17–4.38, *p* = 0.008).	Large sample size. Several disease groups.	Tremor status was by self-report. “Tremor” was not well-defined and it is not the same as ET. There was no adjustment for family size or age of the relatives in PD vs. control families. The statistical design was unsophisticated; the investigators reported proportions rather than using Cox proportional hazards models.
Semchuk and Love (1995) [55]	Canada	Family study	Results the same as Semchuk et al. (1993) [48]	See Semchuk et al. (1993) [48].	See Semchuk et al. (1993) [48]
De Michele et al. (1996) [56]	Italy	Family study	There was a total of 116 PD patients, 116 spouses of PD patients and 116 controls. A positive family history of ET was noted in 19 (16.4%) of 116 PD patients, 6 (5.2%) of 116 spouses of PD patients (OR = 3.59 ^a^, 95% CI = 1.38–9.36, *p* = 0.004) and 7 (6.0%) of 116 controls (OR = 3.05 ^a^, 95% CI = 1.23–7.57, *p* = 0.008).	Two control groups.	Disease status in the relatives was largely by proband report only. The diagnostic criteria for ET were not specified. There was no adjustment for family size or age of the relatives in PD vs. control families.
Taylor et al. (1999) [57]	USA	Family study	Family history of tremor was reported in 12.1% of 140 PD cases and 3.4% of 147 controls (OR = 3.97, 95% CI = 1.17–13.50, *p* = 0.027).	Large sample size. Control group.	Disease status in the relatives was largely by proband report only. The outcome of interest was “tremor” rather than ET. There was no adjustment for family size or age of the relatives in PD vs. control families.
Zorzon et al. (2002) [58]	Italy	Family study	Family history of ET in a first-degree relative was reported in 9 (6.6%) of 136 PD cases and 3 (1.1%) of 272 controls (OR = 10.8, 96% CI = 2.6–43.7, *p* = < 0.0001).	Large sample size. Control group.	Disease status in the relatives was by proband report only. The diagnostic criteria for ET were not specified. There was no adjustment for family size or age of the relatives in PD vs. control families.
Kang et al. (2005) [59]	USA	Family study	In total, 15.4% of 162 PD patients reported a positive family history of ET.	Large sample size.	No control group. Disease status in the relatives was by proband report only. The diagnostic criteria for ET were not specified.
Rocca et al. (2007) [60]	USA	Family study	Relatives of patients with PD had a modest and marginally significantly increased risk of ET compared with control relatives (HR = 1.51, 95% CI 0.95–2.41, *p* = 0.08), with the highest HR seen in relatives of PD patients with younger onset PD (HR = 2.24, 95% CI 1.26–3.98, *p* = 0.006).	Large sample size. Sophisticated statistical design. Control group.	Use of screening interviews. Data from proxies.
Spanaki and Plaitakis (2009) [61]	Crete	Family study	In total, 26 (8.6%) of 277 PD patients vs. 8 (3.2%) of 241 controls reported at least one first-degree relative with ET (OR = 2.83, 95% CI = 1.19–6.92, *p* = 0.015). In total, 41 (2.8%) of 1483 first-degree relatives of PD cases had ET vs. 10 (0.8%) of 1315 first-degree relatives of controls (OR = 3.64, 95% CI = 1.75–7.77, *p* = 0.0001).	Large sample size. Control group. Disease status in many relatives was confirmed by in-person evaluation.	Disease status in some relatives was based on medical record review and surrogate interviews. The statistical design was unsophisticated; the investigators reported proportions and ORs rather than using Cox proportional hazards models.
Costello et al. (2010) [62]	California	Family study	Relatives of individuals with PD had a modestly increased risk of ET, although this increased risk was only marginally significant—45 of 2980 (1.5%) first-degree relatives of PD patients had ET vs. 31 of 2981 (1.0%) first-degree relatives of controls (HR = 1.44, 95% CI -= 0.90–2.29, *p* = 0.13). In several PD subgroups, ET risk was most elevated, including relatives of tremor-dominant PD cases (HR = 1.69, 95% CI 0.99–2.88, *p* = 0.05), relatives of younger-onset PD cases (HR = 2.03, 95% CI = 0.93–4.44, *p* = 0.08) and male relatives (HR = 2.31, 95% CI = 1.13–4.73, *p* = 0.02).	Large sample size. Control group. Sophisticated statistical design.	Diagnosis of ET in relatives was exclusively by proband report.
Fekete et al. (2010) [42]	USA	Family study	Family history of ET-type tremor was identified in 21.5% of PD cases, 6.5% of PSP cases, and 5.0% of CBD cases.	Several disease groups.	Diagnosis of “ET-type tremor” rather than “ET” was ascertained and was exclusively by proband report. Sample size not specified; hence, OR could not be calculated.
Louis et al. (2016) [63]	USA	Family study	A higher percentage of PD than control probands reported relatives with PD ET (11.5% vs. 2.8%, *p* = 0.002). However, the proportion of ET probands and controls probands who reported a relative with PD was similar (10.9% vs. 8.5%, *p* = 0.49).	Large sample size. Control group.	Diagnosis was exclusively by proband report. The statistical design was unsophisticated; the investigators reported proportions rather than using Cox proportional hazards models.

CI = confidence interval, ET = essential tremor, OR = odds ratio, PD = Parkinson’s disease, py = person-years. ^a^ Calculated by Dr. Louis based on data presented in the publication.

## Abbreviations

The following abbreviations were used in this manuscript:

CBD	Corticobasal degeneration
CI	Confidence Interval
ET	Essential tremor
HR	Hazards ration
PD	Parkinson’s disease
PSP	Progressive Supranuclear Palsy
Py	person years
RBD	rapid eye movement sleep behavior disorder
RR	Relative risk
SPECT	Single-photon emission computed tomography
USA	United States of America
